# Psychometric Properties of the South Oaks Gambling Screen Revised for Adolescents in Chinese Adolescent Gamblers

**DOI:** 10.3389/ijph.2022.1605182

**Published:** 2022-11-17

**Authors:** Hui Zhou, Juliet Honglei Chen, Haofeng Ling, Kwok Kit Tong, Anise M. S. Wu

**Affiliations:** ^1^ Department of Psychology, Faculty of Social Sciences, University of Macau, Macao, China; ^2^ Centre for Cognitive and Brain Sciences, Institute of Collaborative Innovation, University of Macau, Macao, China

**Keywords:** screening, health, adolescent, Chinese, psychometric properties, gambling disorder, SOGS-RA

## Abstract

**Objectives:** Adolescent gambling is a public health concern of increasing importance. The lack of comprehensive evaluations on adolescent gambling disorder (GD) assessment tools hinders the timely detection of Chinese adolescents with gambling problems. This study aimed to evaluate the psychometric properties of South Oaks Gambling Screen-Revised for Adolescents (SOGS-RA) and determine its optimal screening cutoff score among Chinese adolescent gamblers to address this gap.

**Methods:** We surveyed 1407 Chinese secondary school students aged 11–19 years in Macao, China, among which 258 past-year gamblers’ data was used for assessing SOGS-RA’s performance in detecting risk for adolescent GD.

**Results:** SOGS-RA displayed satisfactory reliability and validity for assessing probable GD among Chinese adolescent gamblers. Under the *DSM-5* GD framework, we proposed ≥4 as SOGS-RA’s optimal cutoff score of screening for probable GD and further identified 5.8% of past-year gamblers prone to probable GD in the present study.

**Conclusion:** SOGS-RA can provide a reliable and valid assessment of adolescent’ GD risk in the Chinese context, facilitate early identification of probable GD cases, and alleviate the public health concern for Chinese adolescents.

## Introduction

With a long history and widespread presence, adolescent gambling has been increasingly recognized as a significant public health concern worldwide [1]. Compared to adults, adolescents appeared to be more prone to gambling problems [2, 3], especially those who had an early gambling engagement at approximately the age of 10–11 years [4]. Early identification of probable cases at risk for gambling disorder (GD) is the key to offering timely interventions to those adolescents in need and ameliorating potential adversities associated with adolescent GD, which include but are not limited to delinquency, poor academic performance, impaired family relationship, mental health issues, and psychosocial adjustment problems [5, 6].

From a social-environmental perspective, Chinese communities have embedded risk factors associated with the onset and developmental course of gambling problems for adolescents. For instance, social gambling, in the form of ‘mahjong’ or poker, is considered an everyday activity for daily leisure and celebration of special events, such as weddings and festivals, among the Chinese [7]. It is not uncommon for Chinese adults to introduce gambling activities to minors. For example, 81% of parents who participated in a survey conducted in Macao, China (*N* = 311) had reported experiences of teaching their underage children to play gambling games [8]. Such positive attitudes towards social gambling may make gambling activities more accessible to Chinese adolescents and thus predispose them to be more vulnerable to gambling-related problems. Furthermore, the development of the Internet and electronic devices has also provided adolescents with new access to gambling, such as Internet gambling, in addition to social gambling with family and friends. According the study of Wong and So [9], young Internet gamblers might be at greater risk for problem/pathological gambling than their land-based counterparts in Hong Kong, China.

As one of the most widely used tools for evaluating adolescent problem gambling, the South Oaks Gambling Screen–Revised for Adolescents (SOGS-RA) has shown a unidimensional structure and good psychometric properties in terms of internal consistency, test-retest reliability, and criterion-related validity in samples of Italian and Canadian secondary school students [10, 11]. Admittedly, there are other alternatives, less popular assessment instruments of adolescent GD, available other than SOGS-RA, such as the Diagnostic and Statistical Manual of Mental Disorders-IV-Multiple Response-Adapted for Juveniles (DSM-IV-MR-J) [12] and the Canadian Adolescent Gambling Inventory (CAGI) [13]; however, only SOGS-RA and DSM-IV-MR-J were consistently endorsed as the best tools for screening adolescent gambling problems in reviews [3, 14]. The present study chose SOGS-RA over DSM-IV-MR-J because not only more studies have tested and supported SOGS-RA’s psychometric soundness (e.g., 66% versus 24% out of 50 studies) [14], but also SOGS-RA has demonstrated potential for high compatibility with the Chinese context [15–17]. Therefore, it is crucial to bridge the gap between the need to mass screening for at-risk cases of GD among Chinese adolescents and the shortfall of assessment tools for Chinese adolescent GD in this regard.

The first objective of the present study was to test the psychometric properties of SOGS-RA [18] among Chinese adolescents. Although several studies have applied SOGS-RA to assess problem gambling severity among Chinese adolescents [16, 17], no previous study has empirically examined its overall psychometric properties in any Chinese communities. To address this missing link, the present study aims to assess its construct validity, internal consistency, test-retest reliability, and criterion-related validity in the Chinese context. Construct validity was tested with confirmatory factor analysis (CFA) to examine the goodness of fit between the hypothesized unidimensional structure of SOGS-RA and the data acquired from secondary school students in China. The scale’s internal consistency and 2-month test-retest reliability were assessed with Cronbach’s alpha and intraclass coefficient, respectively. Because functioning impairment [19, 20] and impulsivity [21, 22] are two of the most typical correlates of adolescent GD, the current study utilized three gambling-induced functioning impairment indicators and impulsivity to evaluate the criterion-related validity of SOGS-RA and expected positive associations between these indicators and SOGS-RA.

Our second objective was to identify an optimal cutoff point of SOGS-RA, with reference to the *DSM-5* criteria for GD, for screening for probable GD cases among Chinese adolescents. The screening cutoff point of the South Oaks Gambling Screen (SOGS) [23] was proposed based on American Psychiatric Association (APA)’s [24] *DSM-III-R* criteria for pathological gambling. As its adolescent version, SOGS-RA was modified from SOGS and utilized a cutoff score of ≥4 for detecting problem gambling [25]. Although empirical studies have widely applied this standard to study adolescent gambling [21, 26–28], no one has yet evaluated the optimal screening cutoff point of SOGS-RA according to the nine criteria for GD suggested by APA’s [29] *DSM-5*. Such an omission would cast doubts on the screening effectiveness of SOGS-RA in facilitating early detection of probable *DSM-5* GD among adolescents. To bridge this research gap, this study attempted to compare the screening efficacy of SOGS-RA’s potential cutoff points and locate the optimal one for identifying cases prone to *DSM-5* GD.

## Methods

### Participants and Procedures

The present study set the following inclusion criteria: both sexes, Chinese secondary school students, and the capability to read traditional Chinese. With a two-stage sampling method, we first drew a random sample of private and public secondary schools in Macao, China, to participate in the study and then requested each school to randomly select a desirable number of classes from different grades according to the school size. Adolescents in Macao may have more exposure to information related to casino gambling, which has been legalized in Macao only, but not any other regions of China, since 1847 [7]. The parental consent was priorly obtained before informed consent from the student participants were sought. In their classrooms, students were briefed about the research aims, anonymity, voluntary nature, and the right to withdrawal at any time by trained research assistants. Only students who gave written consent to research participation took part in the survey, in which they voluntarily completed an anonymous paper-version questionnaire without any compensation in their classrooms and returned it to the trained research assistant.

From late October to early December 2020, we invited 86 classes from 12 secondary schools in Macao, China, to participate in the survey. We eventually received 1407 valid responses (53.1% male; age = 11–19 years, *M* = 14.50, *SD* = 1.62), of which 274 were past-year gamblers. Because a complete response to all SOGS-RA items is a prerequisite for conducting psychometric assessments and identifying an optimal cutoff point of this scale, we further screened out 16 cases (5.8%) with missing values on SOGS-RA items and generated a sample of 258 cases (55.8% male; age = 11–19 years, *M* = 15.15, *SD* = 1.43) for subsequent data analyses. Additionally, we collected a 2-month follow-up sample of 50 students with past-year gambling experience to evaluate the test-retest reliability of SOGS-RA. After deleting those cases with missing data, we included the data of 31 students (61.3% male; age = 12–18 years, *M* = 15.42, *SD* = 1.23) for computing the 2-month test-retest reliability. We had obtained ethical approval for this study from the affiliated university of the corresponding author and implemented the study following the 1964 Declaration of Helsinki and its later amendments.

### Measures

When developed, four SOGS-RA items assessing 1) lifetime and past-12-month frequency of various gambling activities, 2) greatest amount of money ever gambled in a year, 3) whether parents gamble, and 4) the respondent’s perception that either parent gambled “too much” were designed to be omitted from the scoring formula [18]. We therefore included in the present study only the 12 scoring items of SOGS-RA. With reference to the Chinese version of SOGS [30], a psychologist completed the English-to-Chinese translation of SOGS-RA. Another clinical psychologist conducted a back-translation for further comparison and confirmed the translation accuracy of this Chinese version. All items were written in traditional Chinese, and participants rated each item (e.g., *“Gambled more than planned to”*), except the first one, with dichotomous answers (i.e., 1 = *yes* and 0 = *no*). The first item has a 4-point response scale (i.e., *never, some of the time*, *most of the time,* and *every time*), and the response was recoded into a binary format (i.e., 0 = *never/some of the time* and 1 = *most of the time/every time*) in the scoring phase. A total score was computed, with a higher score representing a severer level of problem gambling among Chinese adolescents.

The 9-item *DSM-5* diagnostic criteria for GD [29] were utilized for receiver operating characteristics (ROC) analysis on SOGS-RA. The participants, who had past-year gambling experience, reported whether they had each GD symptom (e.g., *“Preoccupation with gambling”*) or not in the past 12 months (using the response scale of 1 = *yes* and 0 = *no*). A higher total score represented more significant self-reported GD symptoms. Consistent with the previous study on Chinese people [31, 32], we adopted the cutoff score of ≥4 in this study.

Following Lin et al.’s [33] diagnostic criteria for assessing functioning impairments associated with smartphone addiction, we adapted three functioning impairment indicators for disordered gambling, including 1) consistent or repetitive occurrence of physical or psychological problems (hereinafter *physical or psychological problems*), 2) impairment to study, work, and/or relationship (hereinafter *study, work, and/or relationship impairments*), and 3) apparent psychological distress (hereinafter *psychological distress*). A sample item is *“Have you experienced consistent or repetitive physical or psychological problems due to gambling”*, with a dichotomous response scale (i.e., 1 = *yes* and 0 = *no*) representing the presence or absence of the corresponding functioning impairment induced by gambling.

This study used the 8-item emotion-based rash action subscale from Xue et al.‘s [34] Chinese version of the short UPPS-P Impulsive Behavior Scale (S-UPPS-P) [35] to measure levels of impulsivity when individuals were confronted with positive and negative emotions. The S-UPPS-P has also been validated in Chinese adolescents [36]. Participants rated each item (e.g., *“When I feel bad, I will often do things I later regret in order to make myself feel better now”*) on a 4-point Likert scale, in which 1 = *strongly agree* to 4 = *strongly disagree*. All items were scored in reverse, and a higher total score represented a higher level of impulsive behavior. The Cronbach’s alpha of this scale was 0.77.

Participants were asked to provide information regarding their sex (1 = *male*; 2 = *female*), age, and past-year gambling behaviors in terms of frequency (i.e., from 0 = *never* to 4 = *always*) and monthly monetary expense (in Macanese pataca). Their self-report expense amount was recoded into a 4-point scale during the scoring process, from 1 = *approximately 1.25 USD or below* to 4 = *approximately 62.5 USD or above*.

### Statistical Analysis

The unidimensional structure of SOGS-RA was first tested by CFA using the Lavaan package in R using diagonally weighted least squares estimation [37]. According to Schreiber et al.’s [38] guidance of goodness of model fit for categorical data, we adopted the satisfactory criteria as the comparative fit index (CFI) ≥ 0.95, Tucker-Lewis index (TLI) ≥ 0.96, and root mean square error of approximation (RMSEA) < 0.06. The reliability and criterion-related validity were assessed in SPSS 26. The reliability of SOGS-RA was measured by the KR-20 coefficient and the intraclass correlation coefficient (ICC) for internal consistency and test-retest reliability, respectively. Bivariate correlations of SOGS-RA with gambling-induced functioning impairments and impulsivity were used to assess the criterion-related validity of SOGS-RA. The statistical significance level was set to *p* < 0.05 in all analyses.

The cutoff score of SOGS-RA for screening probable GD cases was also estimated. Firstly, the ROC of the SOGS-RA was analyzed to obtain the *DSM-5* GD-referenced area under the curve (AUC), which was taken as a general indicator of SOGS-RA’s screening efficacy for probable GD. Then, with the *DSM-5* diagnostic criteria for GD classification (self-report score ≥4), the additional screening efficacy indices were computed with DAG_STAT [39], including sensitivity, specificity, positive predictive rate (PPR), negative predictive rate (NPR), Youden’s index, and diagnostic odds ratio (DOR). All screening efficacy indices were taken into account to evaluate the possible cutoff points (i.e., the sensitivity and specificity rates were higher than 75%) [40] and identify the optimal one to maximize the screening efficacy for detecting probable GD cases. Furthermore, the entire sample was divided into probable GD group and non-GD group for further between-group comparison to investigate the discriminant validity of the proposed SOGS-RA screening cutoff point.

## Results

### Gambling Characteristics of the Sample

Among 258 past-year gamblers, the self-report gambling frequency over the past 12 months was 1.6% *always*, 5.4% *often*, 23.6% *sometimes*, and 69.4% *rarely* on the 5-point response scale from 0 = *never* to 5 = *always.* As for monthly gambling expenses, about three-quarters of the respondents (73.3%) spent less than 12.5 USD on gambling activities per month, whereas 10.1% of the respondents had gambled more than 62.5 USD per month. In addition, no significant differences on gambling frequency (*M*_*Rank*_: male = 133.80, female = 122.88, Mann-Whitney *U* = 7445.00, *p* = 0.149) and monthly expenses (*M*_*Rank*_: male = 133.86, female = 126.63, Mann-Whitney *U* = 7868.00, *p* = 0.63) have been found between male adolescents and female adolescents.

### Confirmatory Factor Analysis and Item Endorsement Rate

The one-factor model of SOGS-RA was tested using CFA. The results showed a good model fit, χ^2^ (54) = 86.09, *p* = 0.004, CFI = 0.98, TLI = 0.97, RMSEA = 0.05, 90% CI [0.03, 0.07], with standardized factor loadings ranged from 0.52 to 0.99 (see Table 1), which supported the hypothesized unidimensionality SOGS-RA’s latent structure. As shown in Table 1, the three most salient GD symptoms among Chinese adolescents in our sample were the inability to control one’s gambling (i.e., 26.4%; Item 4), being criticized because of gambling (i.e., 16.7%; Item 5), and feeling bad about one’s gambling involvement (i.e., 16.3%; Item 6).

**TABLE 1:** Standardized factor loadings and endorsement rates of South Oaks Gambling screen revised for adolescents items among Chinese past-year adolescent gamblers (Macao, China, 2020).

South oaks gambling screen revised for adolescents items	Standardized factor loading	Endorsement rate (Yes%)
1. In the past 12 months, how often have you gone back another day to try to win back the money you lost? a	0.75	5.8
2. In the past 12 months when you were betting, have you ever told others you were winning money when you really were not winning?	0.59	7.4
3. Has your betting money, in the past 12 months, ever caused any problems for you such as arguments with family and friends, or problems at school or work?	0.83	3.5
4. In the past 12 months, have you ever gambled more than you had planned to?	0.52	26.4
5. In the last 12 months, has anyone criticized your betting or told you that you had a gambling problem, regardless of whether you thought it was true or not?	0.65	16.7
6. In the past 12 months, have you ever felt bad about the amount you bet, or about what happens when you bet money?	0.67	16.3
7. Have you ever felt, in the past 12 months, that you would like to stop betting money but did not think you could?	0.75	6.2
8. In the last 12 months, have you ever hidden from family or friends any betting slips, I.O.U.s, lottery tickets, money that you’ve won, or other signs of gambling?	0.67	2.7
9. In the past 12 months, have you had money arguments with family or friends that centered on gambling?	0.86	3.5
10. In the past 12 months, have you borrowed money to bet and not paid it back?	0.92	2.7
11. In the past 12 months, have you skipped or been absent from school or work due to betting activities?	0.65	0.4
12. Have you borrowed money or stolen something in order to bet or to cover gambling debts in the last 12 months?	0.99	1.6

^a^ Note: The original 4-point response scale (from *never* to *every time*) of this item was recoded into No [0] = *never/some of the time* and Yes [1] *= most of the time/every time* according to the scoring rule for SOGA-RA.

### Reliability and Criterion-Related Validity

The reliability of SOGS-RA was satisfactorily high in terms of both internal consistency (KR-20 = 0.71) and 2-month test-retest reliability (ICC = 0.77). The criterion-related validity was generally supported as evidenced by the significant correlations of SOGS-RA with physical or psychological problems (*r*_*pb*_ = 0.36, *p* < 0.001), study, work, and/or relationship impairments (*r*_*pb*_ = 0.47, *p* < 0.001), psychological distress (*r*_*pb*_ = 0.47, *p* < 0.001), and impulsivity (*r* = 0.16, *p* < 0.05) in the expected direction.

### Screening Efficacy and Determining the Screening Cutoff Score of SOGS-RA

The ROC analysis showed good efficacy of SOGS-RA (AUC = 0.98) as referenced to the *DSM-5* GD diagnostic criteria **≥** 4. The process of determining the cutoff score of SOGS-RA started with listing all possible cutoff scores with sensitivity and specificity higher than 75% (i.e., points from 2 to 4; see Table 2) as potential candidates. Subsequently, the point of **≥** 4 was chosen as the optimal screening cutoff score of SOGS-RA because of a higher level of sensitivity, specificity, PPR, NPR, Youden’s index, and DOR than the other two possible cutoff points (i.e., 2 and 3). With this cutoff score, the probable GD ratio was 5.8% in our study, higher than the GD proportion estimated by *DSM-5* diagnostic criteria for GD (i.e., 0.8%).

**TABLE 2:** Cutoff points of South Oaks Gambling screen revised for adolescents based on the fifth edition of the diagnostic and statistical manual of mental disorders diagnostic criteria for gambling disorder among Chinese adolescent past-year gamblers (Macao, China, 2020).

Cutoff point (≥)	Sensitivity (%)	Specificity (%)	PPR (%)	NPR (%)	Youden’s index	DOR
1	100.0	55.9	1.7	100.0	0.56	6.32
2	100.0	77.3	3.3	100.0	0.77	16.97
3	100.0	88.7	6.45	100.0	0.89	38.56
**4**	**100.0**	**94.9**	**13.3**	**100.0**	**0.95**	**90.19**
5	50.0	98.1	16.7	99.6	0.48	45.73

Note: PPR, positive predictive rate; NPR, negative predictive rate; DOR, diagnostic odds ratio. The boldfaced line denotes the proposed cutoff score.

According to the cutoff score of SOGS-RA (i.e., **≥** 4), we divided the participants into probable GD group and non-GD group for further between-group comparison to evaluate the discriminant power of this cutoff score. Our results showed that the probable GD group reported significantly higher levels in physical or psychological problems (*r*ϕ = 0.24, *p* < 0.001), study, work, and/or relationship impairments (*r*ϕ = 0.31, *p* < 0.001), psychological distress (*r*ϕ = 0.26, *p* < 0.001), and gambling frequency (*M*_*Rank*_: probable GD = 180.60, Non-GD = 126.35, Mann-Whitney *U* = 1056.00, *p* < 0.01) than the non-GD group. Consistent with the results shown in the overall past-year gamblers, the three most rated SOGS-RA items remained to be uncontrollability over gambling (93.3%), receiving criticism because of gambling (80.0%), and feeling bad about one’s gambling involvement (86.7%) among the probable GD gamblers, though the rates were significantly higher.

## Discussion

In the light of two objectives, the present study is the first empirical study that provides a comprehensive psychometric evaluation of SOGS-RA regarding its dimensionality, reliability, validity, and screening efficacy among Chinese adolescents and proposes an optimal screening cutoff point for problem adolescent gamblers in China. Regarding the first study objective, our results substantiated the hypothesized, satisfactory psychometric properties of SOGS-RA in the Chinese context. Consistent with the previous validation studies of SOGS-RA among adolescents in other countries (e.g., Italy and Canada) [10, 11], our data fitted the conceptualized unidimensional structure of the SOGS-RA well. We also found that SOGS-RA provides a reliable assessment of Chinese adolescents’ gambling severity, as evidenced by its acceptable internal consistency and 2-month test-retest reliability in the present study. Furthermore, in line with our expectations and previous findings [19, 21], the good criterion-related validity of SOGS-RA was substantiated by its significantly positive correlation with gambling-induced functioning impairments and impulsivity.

As for the second study objective, we first confirmed that SOGS-RA presented good screening efficacy for identifying cases that fit the diagnostic criteria of *DSM-5* GD (AUC = 0.98). We further identified an optimal cutoff score of SOGS-RA as **≥** 4, with high sensitivity and specificity (i.e., 100.0% and 94.9%, respectively), to screen our Chinese adolescent past-year gamblers with high risk for probable *DSM-5* GD satisfactorily. The exact cutoff score of **≥** 4 was widely adopted worldwide (e.g., Italy, Spain, and South Korea) [26–28] since Winters et al. [25] first proposed it in their original SOGS-RA studies based on teenager data from the United States; however, seldom did researchers examine the screening efficacy of this cutoff point in other regions ever since. Our present study has lent extra empirical data to a cross-regional analysis of the cutoff points of SOGS-RA in addition to its handful of precedent studies. Although Boudeau and Poulin’s [11] study has also proposed a cutoff point of **≥** 4 for Canadian adolescent gamblers, they have reported significantly lower sensitivity rates of 58.9–62.0% than ours (i.e., 100.0%) and comparable specificity rates of 95.8–96.4% as ours (i.e., 94.9%). These apparent differences in how the exact cutoff score performed among adolescent gamblers across regions not only underscored a good screening efficacy of SOGS-RA with the currently identified cutoff score in our present sample, but also highlighted the necessity and importance of determining an optimal cutoff score regionally to ensure a cost-effective screening of probable GD cases.

When applying this cutoff score of **≥** 4 in the present sample, probable GD gamblers reported significantly more functioning impairments (i.e., physical or psychological problems, study, work, and/or relationship impairments, and psychological distress) and gambling engagement than their non-GD counterparts, implying an adequate screening efficacy of this optimal cutoff score for Chinese adolescent gamblers. Admittedly, the PPR associated with this cutoff score is relatively low (i.e., 13.3%) in the present sample. The PPR reflects the extent to which the positive test cases are confirmed positive by the gold standard [41]. It is expected and not uncommon to see a relatively low PPR among screening tools that aim to increase the capacity to identify as many probable problematic cases as possible. Similar to our present finding, another SOGS validation study among Chinese also reported a relatively low PPR of 27.0% [42], which may indicate a shared feature between SOGS and its related version (i.e., SOGS-RA in our case) for Chinese communities. Nevertheless, the cutoff score of ≥4 displayed excellent hits of positive and negative cases (i.e., 100% sensitivity and 94.9% specificity) that were identified by the *DSM-5* diagnostic criteria for GD in the present sample; therefore, taking into account all the screening efficacy indices, we considered SOGS-RA, pairing with this optimal cutoff score, to be an effective screening tool for detecting probably GD cases among Chinese adolescents.

Among the 12 items from SOGS-RA, three items (i.e., Item 4 [uncontrollability over gambling], Item 5 [receiving criticism because of gambling], and Item 6 [feeling bad about one’s gambling involvement]) consistently showed the highest endorsement rates in both the overall past-year gambler sample (i.e., 16.3%–26.4%) and the probable GD subsample (i.e., 80.0–93.3%). The wide prevalence of these three symptoms, especially the significantly heightened rates among the probable GD gamers than the gamblers in general, indicated the value of applying these three indicators to facilitate early detection of gamblers who might be at a greater risk for developing GD and the potential of designing tailored intervention strategies based on these indicators. It is also worth noting the apparent difference between those who received criticism because of gambling (i.e., 16.7%) and those who had money arguments about gambling with family and friends (i.e., 3.5%) among the past-year gamblers in our data; this noticeable difference may imply that the “criticism” period can be a good window for preventing further development of adolescent gambling problems before those criticisms transformed into money arguments. Considering the generally high social acceptance of gambling for leisure in China [7, 43], educational campaigns shall be developed for the general public to enhance the awareness of indicators of disordered gambling among adolescents and enable more parents, teachers, and even adolescent peers to grasp the window of criticism for effective GD prevention.

The present study is limited in several aspects. First, our pioneering findings on SOGA-RA among Chinese secondary school adolescents may not be generalized to those not in the schooling system (e.g., the drop-outs). Future studies may consider replicating the current evaluation procedures for SOGS-RA with a more general, community-dwelling adolescent sample, including those adolescent minorities who cannot be reached at school, across regions in China. Second, we did not include a clinical diagnostic procedure to identify a diagnostic cutoff score in the present study. SOGS-RA can be applied to a two-stage epidemiological study in the future, in which probable GD cases are screened out with the current screening cutoff score (i.e., ≥4) at the first stage, while a diagnostic cutoff score can be determined based on the clinical diagnostic results. Third, the current data is inevitably subject to self-report bias due to the survey’s self-report nature. We call for subsequent studies to collect additional, preferably multi-module (e.g., behavioral observation and experiment), data from multiple sources (e.g., parents) for supplementary purposes.

To conclude, the current empirical study made the first step to comprehensively evaluate the psychometric properties of SOGS-RA and identify its optimal cutoff scores to screen for probable *DSM-5* GD among Chinese adolescent gamblers. Our findings supported that SOGS-RA is a valid and reliable assessment tool for evaluating Chinese adolescents’ gambling problems. The proposed optimal screening cutoff score of **≥** 4 displayed satisfactory screening efficacy in detecting probable adolescent GD cases. We recommend using the SOGS-RA to facilitate the mass screening of GD risk among Chinese adolescents in school and community settings as an effective approach to ameliorate public health concerns for adolescent gambling. Subsequent studies may also consider applying it at the first screening stage in a two-stage epidemiological study and further identifying a diagnostic cutoff score at the second stage.

## References

[1] Calado F Alexandre J Griffiths MD. Prevalence of Adolescent Problem Gambling: A Systematic Review of Recent Research. J Gambl Stud (2017) 33:397–424. 10.1007/s10899-016-9627-5 27372832 PMC5445143

[2] Burge AN Pietrzak RH Petry NM. Pre/Early Adolescent Onset of Gambling and Psychosocial Problems in Treatment-Seeking Pathological Gamblers. J Gambl Stud (2006) 22:263–74. 10.1007/s10899-006-9015-7 16816990

[3] Volberg RA Gupta R Griffiths MD Ólason DT Delfabbro P. An International Perspective on Youth Gambling Prevalence Studies. Int J Adolesc Med Health (2010) 22:3–38. 10.1515/IJAMH.2010.22.1.3 20491416

[4] Derevensky JL Gupta R. Prevalence Estimates of Adolescent Gambling: A Comparison of the SOGS-RA, DSM-IV-J, and the GA 20 Questions. J Gambl Stud (2015) 2:4–12. 10.1097/02024458-201509000-00002 14634314

[5] Farhat LC Roberto AJ Wampler J Steinberg MA Krishnan-Sarin S Hoff RA Self-injurious Behavior and Gambling-Related Attitudes, Perceptions and Behaviors in Adolescents. J Psychiatr Res (2020) 124:77–84. 10.1016/j.jpsychires.2020.02.016 32126363

[6] Frisone F Settineri S Sicari PF Merlo EM. Gambling in Adolescence: a Narrative Review of the Last 20 Years. J Addict Dis (2020) 38:438–57. 10.1080/10550887.2020.1782557 32634072

[7] Wu AMS Lau JTF. Gambling in China: Socio-Historical Evolution and Current Challenges. Addiction (2015) 110:210–6. 10.1111/add.12710 25238131

[8] So EMT Lao YMP Wong ILK. Macau Parents’ Perceptions of Underage Children’s Gambling Involvement. Asian J Gambl Issues Public Health (2017) 7:1–10. 10.1186/s40405-017-0021-8 28316902 PMC5334386

[9] Wong ILK So EMT. Internet Gambling Among High School Students in Hong Kong. J Gambl Stud (2014) 30:565–76. 10.1007/s10899-013-9413-6 24190276 PMC4141147

[10] Anselmi P Colledani D Andreotti A Robusto E Fabbris L Vian P An Item Response Theory-Based Scoring of the South Oaks Gambling Screen–Revised Adolescents. Assessment (2021) 29:1381–91. 10.1177/10731911211017657 34036842

[11] Boudreau B Poulin C. The South Oaks Gambling Screen-Revised Adolescent (SOGS-RA) Revisited: A Cut-point Analysis. J Gambl Stud (2007) 23:299–308. 10.1007/s10899-006-9039-z 17180721

[12] Fisher S. Developing the DSM-IV-DSM-IV Criteria to Identify Adolescent Problem Gambling in Non-clinical Populations. J Gambl Stud (2000) 16:253–73. 10.1023/a:1009437115789 14634315

[13] Wiebe J Wynne H Stinchfield R Tremblay J. The Canadian Adolescent Gambling Inventory (CAGI) Phase II Final Report. Calgary, Canada: University of Calgary (2007). Available at: http://dspace.ucalgary.ca/bitstream/1880/48157/1/CAGI_Phase_2_Report-English.pdf .

[14] Edgren R Castrén S Mäkelä M Pörtfors P Alho H Salonen AH. Reliability of Instruments Measuring At-Risk and Problem Gambling Among Young Individuals: A Systematic Review Covering Years 2009–2015. J Adolesc Health (2016) 58:600–15. 10.1016/j.jadohealth.2016.03.007 27151759

[15] Chen JH Wu AMS Tong K. Evaluation of Psychometric Properties of the Inventory of Gambling Motives, Attitudes and Behaviors Among Chinese Adolescents. Int J Ment Health Addict (2015) 13:361–75. 10.1007/s11469-014-9536-8

[16] Wong SSK. Effects of Outcome Expectancies on Chinese Adolescents’ Gambling Intention. J Gambl Commer Gaming Res (2016) 1:34–46. 10.17536/jgcgr.2016.003

[17] Wong SSK Tsang SKM. Validation of the Chinese Version of the Gamblers’ Belief Questionnaire (GBQ-C). J Gambl Stud (2012) 28:561–72. 10.1007/s10899-011-9286-5 22160622

[18] Winters KC Stinchfield RD Fulkerson J. Toward the Development of an Adolescent Gambling Problem Severity Scale. J Gambl Stud (1993) 9:63–84. 10.1007/BF01019925

[19] Hodgins DC. Reliability and Validity of the Sheehan Disability Scale Modified for Pathological Gambling. BMC Psychiatry (2013) 13:177. 10.1186/1471-244X-13-177 23816165 PMC3701601

[20] Müller KW Wölfling K Dickenhorst U Beutel ME Medenwaldt J Koch A Recovery, Relapse, or Else? Treatment Outcomes in Gambling Disorder from a Multicenter Follow-Up Study. Eur Psychiatry (2017) 43:28–34. 10.1016/j.eurpsy.2017.01.326 28365465

[21] Canale N Scacchi L Griffiths MD. Adolescent Gambling and Impulsivity: Does Employment during High School Moderate the Association? Addict Behav (2016) 60:37–41. 10.1016/j.addbeh.2016.04.001 27085156

[22] Secades-Villa R Martínez-Loredo V Grande-Gosende A Fernández-Hermida JR. The Relationship between Impulsivity and Problem Gambling in Adolescence. Front Psychol (2016) 7:1931. 10.3389/fpsyg.2016.01931 28008322 PMC5143594

[23] Lesieur HR Blume SB. The South Oaks Gambling Screen (SOGS): a New Instrument for the Identification of Pathological Gamblers. Am J Psychiatry (1987) 144:1184–8. 10.1176/ajp.144.9.1184 3631315

[24] American Psychiatric Association. Diagnostic and Statistical Manual of Mental Disorders. 3th ed. Washington, DC: The American Psychiatric Association (1987).

[25] Winters KC Stinchfield RD Kim LG. Monitoring Adolescent Gambling in Minnesota. J Gambl Stud (1995) 11:165–83. 10.1007/BF02107113 24233428

[26] Canale N Griffiths MD Vieno A Siciliano V Molinaro S. Impact of Internet Gambling on Problem Gambling Among Adolescents in Italy: Findings from a Large-Scale Nationally Representative Survey. Comput Hum Behav (2016) 57:99–106. 10.1016/j.chb.2015.12.020

[27] González-Roz A Fernández-Hermida JR Weidberg S Martínez-Loredo V Secades-Villa R. Prevalence of Problem Gambling Among Adolescents: A Comparison across Modes of Access, Gambling Activities, and Levels of Severity. J Gambl Stud (2017) 33:371–82. 10.1007/s10899-016-9652-4 27785589

[28] Kim Y Lee S Park A Lee J. Screening Performance of the Korean Version of the Gambling Problem Severity Subscale of the Canadian Adolescent Gambling Index (CAGI GPSS). Int J Ment Health Addict (2022) 20:1083–93. 10.1007/s11469-020-00427-5

[29] American Psychiatric Association. Diagnostic and Statistical Manual of Mental Disorders. 5th ed. Washington, DC: The American Psychiatric Association (2013).

[30] Tang CS Wu AMS Tang JYC Yan ECW. Reliability, Validity, and Cut Scores of the South Oaks Gambling Screen (SOGS) for Chinese. J Gambl Stud (2010) 26:145–58. 10.1007/s10899-009-9147-7 19680794 PMC2953627

[31] Chui WY Lee SK Mok YL Tsang CK. The Diagnostic Criteria of Gambling Disorder of DSM-5 in Chinese Culture: By Confirmatory Factor Analysis (CFA) and Item Response Theory (IRT). In: Leung, M-T Tan L-M, editors. Applied Psychology Readings. Singapore: Springer Singapore (2018). p. 73–86. 10.1007/978-981-10-8034-0

[32] Wu AMS Lai MHC Tong K-K. Gambling Disorder: Estimated Prevalence Rates and Risk Factors in Macao. Psychol Addict Behav (2014) 28:1190–7. 10.1037/a0037603 25134026

[33] Lin YH Chiang CL Lin PH Chang LR Ko C-H Lee YH Proposed Diagnostic Criteria for Smartphone Addiction. PLOS ONE (2016) 11:e0163010. 10.1371/journal.pone.0163010 27846211 PMC5112893

[34] Xue Z Hu Y Wang J Huang L Liu W Sun F. Reliablity and Validity of the Short Version of UPPS-P Impulsive Behavior Scale in College Students. Chin J Clin Psychol (2017) 25:662–6.

[35] Cyders MA Littlefield AK Coffey S Karyadi KA. Examination of a Short English Version of the UPPS-P Impulsive Behavior Scale. Addict Behav (2014) 39:1372–6. 10.1016/j.addbeh.2014.02.013 24636739 PMC4055534

[36] Wang Y Long J Liu Y Liu T Billieux J. Psychometric Properties of the Chinese SUPPS-P Impulsive Behavior Scale: Factor Structure and Measurement Invariance across Gender and Age. Front Psychiatry (2020) 11:529949. 10.3389/fpsyt.2020.529949 33329077 PMC7710909

[37] Li CH. Confirmatory Factor Analysis with Ordinal Data: Comparing Robust Maximum Likelihood and Diagonally Weighted Least Squares. Behav Res Methods (2016) 48:936–49. 10.3758/s13428-015-0619-7 26174714

[38] Schreiber JB Nora A Stage FK Barlow EA King J. Reporting Structural Equation Modeling and Confirmatory Factor Analysis Results: A Review. J Educ Res (2006) 99:323–38. 10.3200/JOER.99.6.323-338

[39] Mackinnon A. A Spreadsheet for the Calculation of Comprehensive Statistics for the Assessment of Diagnostic Tests and Inter-rater Agreement. Comput Biol Med (2000) 30:127–34. 10.1016/S0010-4825(00)00006-8 10758228

[40] Chen JH Zhang MX Ko C-H Tong KK Yu SM Sou EKL The Development of a Screening Tool for Chinese Disordered Gamers: The Chinese Internet Gaming Disorder Checklist (C-IGDC). Int J Environ Res Public Health (2020) 17:3412. 10.3390/ijerph17103412 32422914 PMC7277076

[41] Cicchetti DV. Guidelines, Criteria, and Rules of Thumb for Evaluating Normed and Standardized Assessment Instruments in Psychology. Psychol Assess (1994) 6:284–90. 10.1037/1040-3590.6.4.284

[42] Tang CS Wu AMS. Screening for College Problem Gambling in Chinese Societies: Psychometric Properties of the Chinese Version of the South Oaks Gambling Screen (C-SOGS). Int Gambl Stud (2009) 9:263–74. 10.1080/14459790903348194

[43] Chiu EY-W Woo K. Problem Gambling in Chinese American Adolescents: Characteristics and Risk Factors. Int J Ment Health Addict (2012) 10:911–22. 10.1007/s11469-012-9387-0

